# High-throughput UAV-based rice panicle detection and genetic mapping of heading-date-related traits

**DOI:** 10.3389/fpls.2024.1327507

**Published:** 2024-03-06

**Authors:** Rulei Chen, Hengyun Lu, Yongchun Wang, Qilin Tian, Congcong Zhou, Ahong Wang, Qi Feng, Songfu Gong, Qiang Zhao, Bin Han

**Affiliations:** ^1^ National Center for Gene Research, Key Laboratory of Plant Design/National Key Laboratory of Plant Molecular Genetics, Center for Excellence in Molecular Plant Sciences, Chinese Academy of Sciences, Shanghai, China; ^2^ University of the Chinese Academy of Sciences, Beijing, China; ^3^ Center for Excellence in Molecular Plant Sciences, Chinese Academy of Sciences, Shanghai, China

**Keywords:** *Oryza sativa*, UAV, objective detection, panicle, heading date, QTL

## Abstract

**Introduction:**

Rice (*Oryza sativa*) serves as a vital staple crop that feeds over half the world's population. Optimizing rice breeding for increasing grain yield is critical for global food security. Heading-date-related or Flowering-time-related traits, is a key factor determining yield potential. However, traditional manual phenotyping methods for these traits are time-consuming and labor-intensive.

**Method:**

Here we show that aerial imagery from unmanned aerial vehicles (UAVs), when combined with deep learning-based panicle detection, enables high-throughput phenotyping of heading-date-related traits. We systematically evaluated various state-of-the-art object detectors on rice panicle counting and identified YOLOv8-X as the optimal detector.

**Results:**

Applying YOLOv8-X to UAV time-series images of 294 rice recombinant inbred lines (RILs) allowed accurate quantification of six heading-date-related traits. Utilizing these phenotypes, we identified quantitative trait loci (QTL), including verified loci and novel loci, associated with heading date.

**Discussion:**

Our optimized UAV phenotyping and computer vision pipeline may facilitate scalable molecular identification of heading-date-related genes and guide enhancements in rice yield and adaptation.

## 1 Introduction

*Oryza sativa* is a staple food crop that feeds billions of people worldwide. Optimizing rice yield is critical for global food security, and heading date - the transition from vegetative to reproductive growth - is a key factor determining yield potential. However, traditional manual phenotyping methods for obtaining rice heading-date-related traits are extremely labor-intensive, time-consuming, error-prone, and insufficient for large-scale phenotyping.

Recent advances in computer vision offer transformative potential for fully automatic, high-throughput, and accurate estimation of heading-date-related traits from digital images. Object detection models have proven highly effective for localizing and counting objects in natural images. Leading approaches fall into two main categories: two-stage detectors like Faster R-CNN (Ren et al., 2017) that are accurate but slow, and one-stage detectors such as YOLO (Redmon et al., 2016) that are fast but can struggle with small objects. However, recent advancements in one-stage detectors have narrowed down this accuracy gap, especially in the YOLO family. Newer transformer-based approaches like DETR (Carion et al., 2020) remove hand-designed components like NMS but suffer from convergence issues. Subsequent works have addressed this problem, making the DETR series an attractive model choice overall.

Several studies have already applied these cutting-edge models for analyze rice panicles for traits related to heading date and yield. For instance, Zhou et al. proposed a pipeline using YOLOv5, DeepSORT for tracking identical panicles over time-series images and quantifying the effects of nitrogen on flowering duration and timing (Zhou et al., 2023). The improved Cascade R-CNN is used to detect rice panicles and recognize growth stages from smartphone images under complex field conditions (Tan et al., 2023). The estimated heading dates by counting flowering panicle regions in ground images under an indirectly image classification manner is also performed (Desai et al., 2019). A lightweight model called TinyCCNet for rice panicle segmentation in UAV images is developed, showing potential for agricultural UAVs with limited computing resources (Ramachandran and K.S., 2023). The Res2Net model has been used to classify growth stages and partial least squares regression to estimate heading date from UAV time series images, achieving high accuracy (Lyu et al., 2023). Overall, these studies demonstrate deep learning and computer vision techniques enable accurate, automatic analysis of panicle development from both aerial and ground-based imagery.

However, some obstacles persist in applying off-the-shelf detectors to new specialized domains like panicle counting. Large annotated image datasets are imperative for training high-performing models, but expensive and time-consuming to obtain for niche applications. Different model architectures are often compared only on generic datasets like COCO (Lin et al., 2015), rather than domain-specific tasks like panicle counting. Finally, optimal models for a given application are unclear.

In this paper, we leveraged UAV high-throughput aerial image combined with a semi-automatic annotation workflow to systematically evaluate various state-of-the-art detectors on rice panicle counting. Our comparative analysis identified YOLOv8-X as the top-performing model for our specific application. Subsequently, we utilized YOLOv8-X to extract multiple heading-date-related traits from UAV time-series images with high throughput and accuracy. With these obtained traits, we were able to identify reliable genetic variants using QTL mapping. Some of these variants were consistent with previously published studies, while others facilitated the exploration of novel candidate genes. Our optimized UAV phenotyping and deep learning pipeline helps overcome key limitations, enabling scalable dissection of the genetic basis of rice heading-date-related traits. All relevant code can be accessed at https://github.com/r1cheu/phenocv.

## 2 Materials and methods

### 2.1 Rice planting and field image collection

Derived from the crossing of Nipponbare (*Oryza sativa* ssp. *japonica*) and 93–11 (*Oryza sativa* ssp. *indica*), a total of 294 RILs of rice (Huang et al., 2010) were cultivated in Ling Shui, Hainan province at an 18-degree north latitude. The rice was sown in plots measuring 2×1.1m, accommodating 18 plants per plot.

During the rice growth process in 2023, a total of 42 aerial flights were conducted using the DJI Matrice M300 equipped with the ZENMUSE H20 (DJI, Shenzhen, China), which integrated a 20-megapixel zoom camera. Operating at a flight altitude of 18 meters, H20 effectively utilizes its 10x zoom capability to capture clear and detailed imagery of each individual rice panicle within the expansive paddy field.

### 2.2 Locating plot region

The original images captured by the H20 centered on an individual plot but covered a larger area. Therefore, as a preprocessing step, we extracted the region that only included the central plot from each original image. We first calculated the expected plot width and length based on a known planting density (30cm between plants, 50cm between plots). We used 3800 × 2000 pixels in this work. Next, we binarized the images using OTSU (Otsu, 1979) threshold with the color index of vegetation (CIVE) (Equation 1) (Kataoka et al., 2003). Then, the numbers of white pixels (representing vegetation) per row/column were calculated. The result was smoothed by moving average with a window size of 100. Finally, we defined the row/column that contained the fewest white pixels as the boundary of the plot, since the boundary should contain the minimum number of plant pixels (Equations 2, 3).

**Figure 1:**
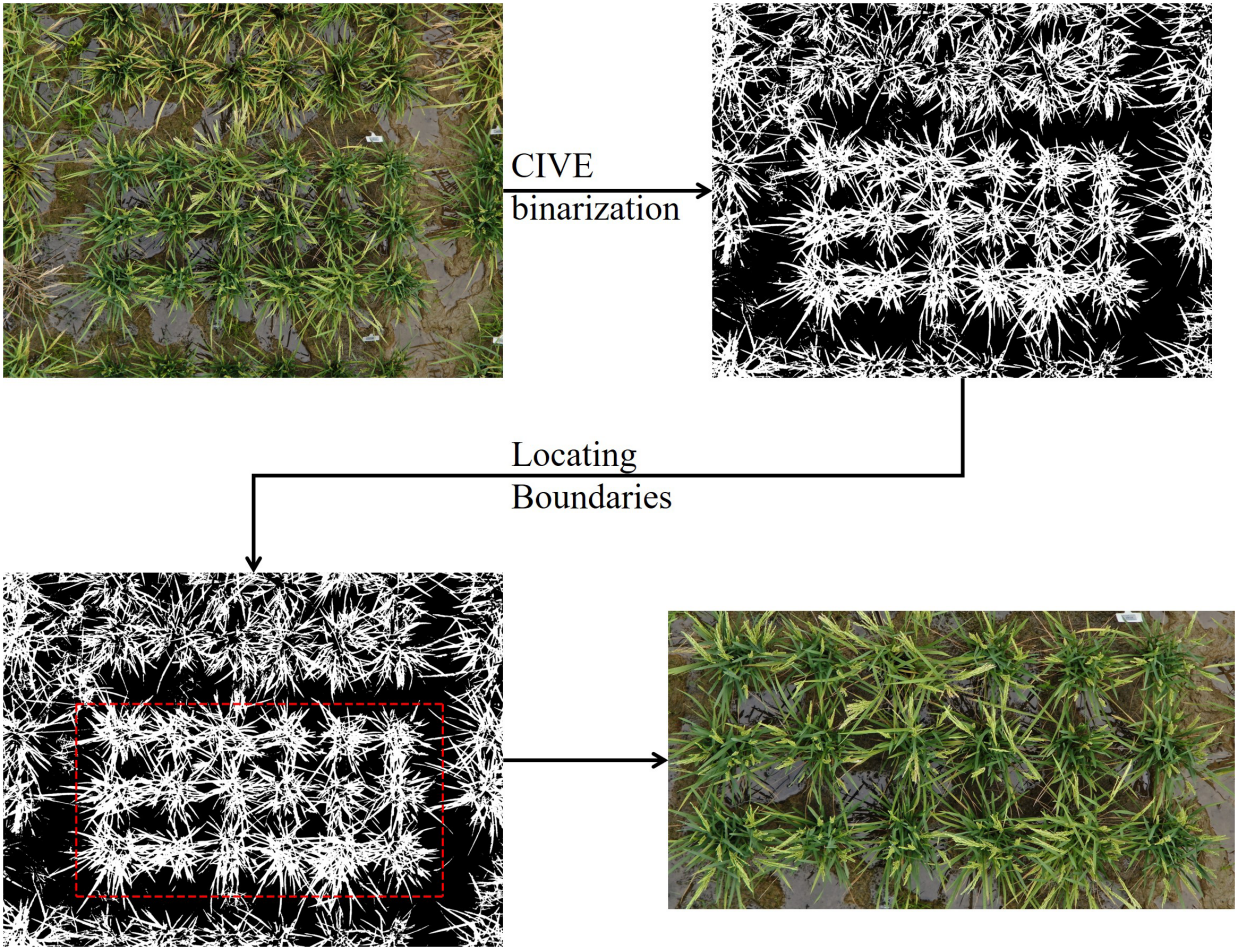
Plot extraction workflow. Follow the direction of arrow, the original UAV image (top left) was first binarized using CIVE index and OSTU’s thresholding. Next, under a fixed box width of 3800 and height of 2000, the box was moved over the entire image to found the row/column containing the fewest white pixels, thus, locating the boundary. Finally, the plot was cropped from the original image.


The Locating workflow was implemented in Python using the NumPy and OpenCV libraries and is described in **Figure 1**.

(1) 0.441×R−0.811×G+0.385×B+18.78754

(2) $$Row\;of\;Plot=min\left(Row_{i}+Row_{i+2000}\right)$$

(3) $$Column\;of\;Plot=min\left(Col_{i}+Col_{i+3800}\right)$$

Where R, G and B are the pixel values for the corresponding red, green, and blue channels. *Row_i_* denotes the count of white pixels in the i-th row.

### 2.3 Annotation workflow

In the annotation workflow, to reduce labor costs and accelerate annotations, we utilized the Label Studio interface with the Segment Anything Model (SAM) as the inference backend. SAM can precisely label a panicle using a single-point prompt, thereby allowing for the creation of bounding box around panicle with just one click.

**Figure 2:**
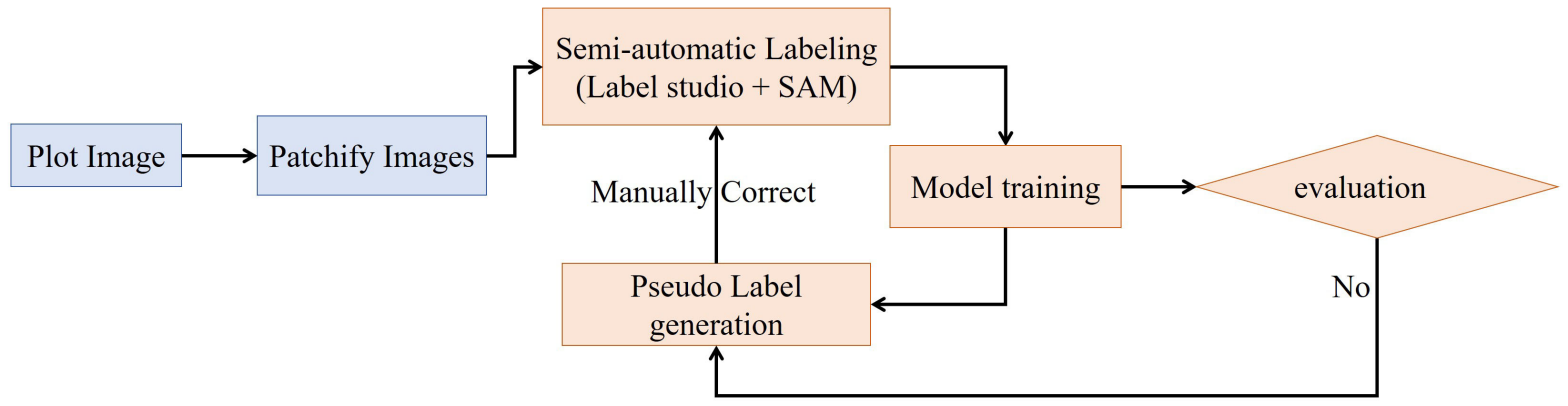
Semi-automatic annotation workflow. The workflow begins with plot images which are patchified into smaller sub-images. These patches undergo semi-automatic labeling using Label Studio interfaced with the SAM model for automated suggestions. The labeled sub-images are used to train a model, which is evaluated to determine if performance is sufficient. If not, the model generates pseudolabels on unlabeled data, which re-enters the semi-automatic labeling stage. When the model evaluation is acceptable, the loop breaks and the final model is produced.

The general annotation workflow is illustrated in **Figure 2**. Initially, we used a sliding window with the shape of 1000×1000 pixels and a stride of 1000×1000 pixels to divide the 3800×2000 plot images into smaller subimages of 1000 × 1000 pixels. Subsequently, we iterated between model-generated pseudo-labeling, human correction, and model retraining until the dataset was fully labeled or the model’s performance met our requirements. This iterative process began with the training of a Faster R-CNN model using approximately 50 labeled images.

In total, we annotated 1852 images and randomly divided them into three datasets with an 8:1:1 ratio. More specifically, we allocated 1530 images for the training set, 161 for the validation set, and another 161 for the test set. Additionally, within the test set, we selected both early-stage and late-stage panicles, creating two subtest sets to ensure a thorough evaluation.

### 2.4 Prediction workflow

**Figure 3:**
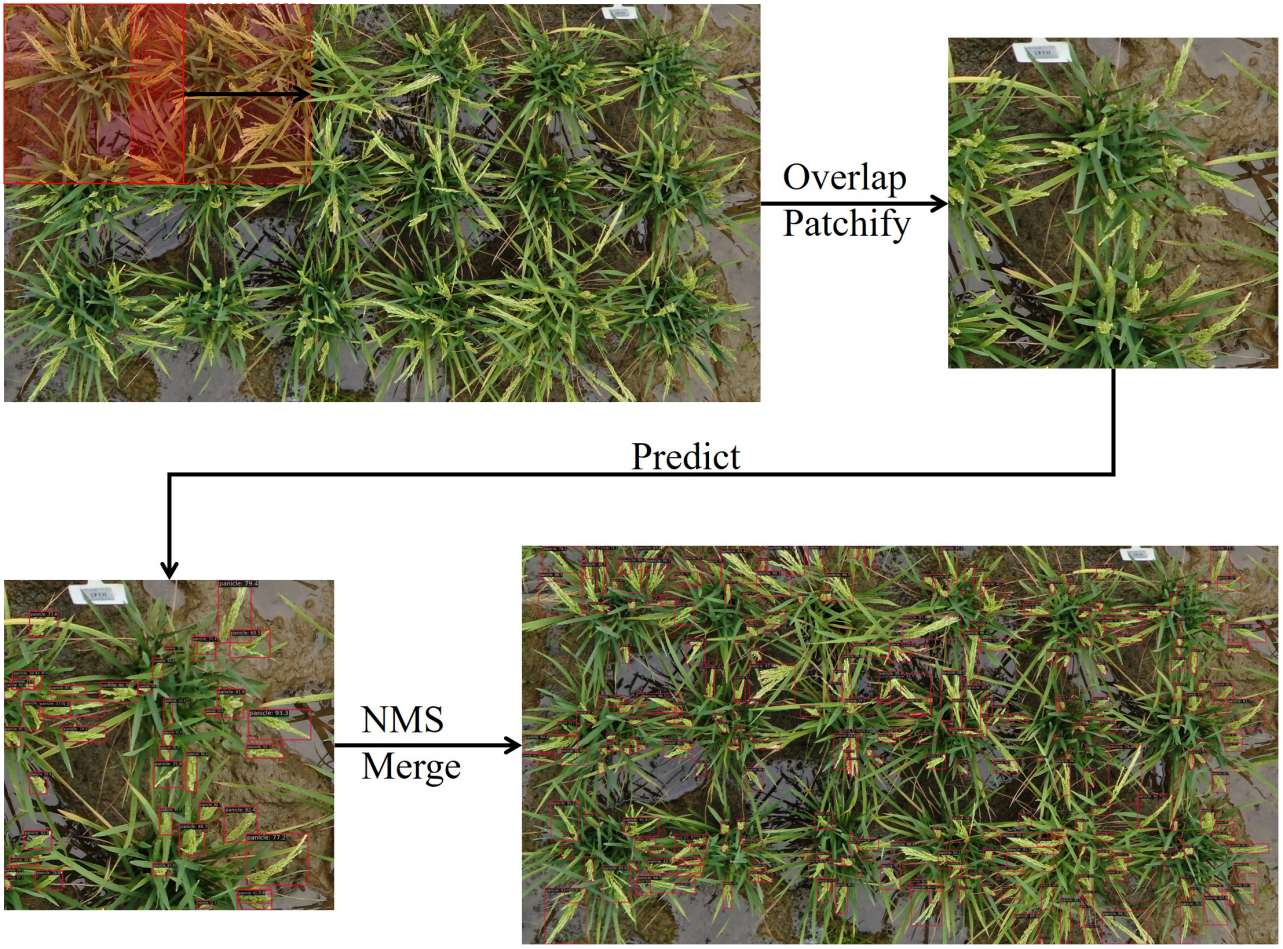
Predicting panicle counts from plot images using overlap sliding window approach. Follow the direction of arrow, the plot image was divide into smaller sub-image using a slide window approach. The sub-images were then fed into objective detection model to predict the location of panicles. Subsequently, the predictions from all sub-images were merged using non maximum suppression to remove the redundant prediction.

The prediction workflow also commenced from the plot image as depicted in **Figure 3**. To begin with, each plot image was split into overlapping sub-images with an overlap ratio of 0.25 and window size of 1000 × 1000 pixels. Next, the model detected panicles within each sub-image. Lastly, the predictions from the same plot image were merged by employing non-maximum suppression with a threshold of 0.25.

The workflow was implemented in Python using Sahi (Akyon et al., 2022), Pytorch, TorchVision, OpenCV (Bradski, 2000), and NumPy (Harris et al., 2020).

### 2.5 Model experimental settings

In general, we followed the default training strategies provided by the MMdetection (Chen et al., 2019) and Ultralvtics (Jocher et al., 2023) libraries, which are highly recommended, optimized, and consistently delivered stable performance. The software environments utilized in this paper include Python 3.9, PyTorch 2.0.1, CUDA 11.8, MMdetection v3.10 and Ultralvtics v8.0.158. All the models were trained on 8 NVIDIA A40 GPUs.

#### 2.5.1 Models

We investigated various objective detection models, including Faster R-CNN (Ren et al., 2017), Cascade R-CNN (Cai and Vasconcelos, 2019), YOLO v5 (Jocher, 2020), YOLO v8 (Jocher et al., 2023), RT-DETR (Lv et al., 2023), DINO (Zhang et al., 2023) with different backbones and model sizes, as outlined in **Table 1**. The implementations of Faster R-CNN, Cascade R-CNN, and DINO utilized the MMdetection library, while the YOLO series and RT-DETR were implemented using the Ultralytics library. All the models were initialized with pretrained weights provided in respective library.

**Table 1:** Performance of detecors on early heading stage, later heading stage, and full test set.

Model	Test				Early		Late	
	mAP_50:5:95_	AP_50_	R^2^	RMSE	R^2^	RMSE	R^2^	RMSE
Faster RCNN-R50	0.571	0.868	0.907	3.894	0.957	2.687	0.821	4.818
Faster RCNN-R101	0.568	0.865	0.900	4.026	0.952	2.833	0.811	4.950
Faster RCNN-CN-t	0.596	0.887	0.818	5.442	0.921	3.638	0.664	6.797
Cascade RCNN-R50	0.588	0.866	0.880	4.416	0.941	3.152	0.775	5.402
Cascade RCNN-R101	0.588	0.865	0.873	4.545	0.931	3.387	0.769	5.474
Cascade RCNN-CN-t	0.618	0.880	0.805	5.636	0.926	3.507	0.604	7.175
YOLOv5-n	0.613	0.875	0.912	3.794	0.948	2.950	0.845	4.490
YOLOv5-m	0.667	0.895	0.908	3.862	0.966	2.378	0.813	4.930
YOLOv5-x	0.675	0.898	0.906	3.918	**0.966**	**2.401**	0.807	5.006
YOLOv5-n-P6	0.660	0.892	0.920	3.618	0.950	2.889	0.862	4.230
YOLOv5-m-P6	0.673	0.892	0.922	3.574	0.965	2.429	0.848	4.441
YOLOv5-x-P6	0.677	0.899	0.923	3.531	0.962	2.526	0.857	4.316
YOLOv8-n	0.621	0.879	0.918	3.653	0.952	2.846	0.856	4.318
YOLOv8-m	0.666	0.893	0.921	3.590	0.955	2.744	0.859	4.285
YOLOv8-x	0.674	0.897	**0.927**	**3.442**	0.963	2.477	**0.864**	**4.200**
RT-DETR-l	0.630	0.887	-0.389	15.041	0.499	9.156	-1.850	19.245
DINO-R50	0.612	0.885	0.770	6.118	0.910	3.881	0.538	7.751
DINO-Swim-L	**0.677**	**0.914**	0.818	5.545	0.913	3.797	0.655	6.697

Where R50, R101, CN-t, Swim-L, stand for ResNet50 (He et al., 2016), ResNet101, ConvNext-Tiny (Liu et al., 2022), Swim Transformer-Large (Liu et al., 2021). All RCNN models use Feature Pyramid Networks (Ren et al., 2017). P6 represents six stages in the backbone and uses the image size of 1280 × 1280 pixels as inputs, while other YOLO models use the 640 × 640 pixels image as inputs. Bold indicates that the value is the best metric value in this column.

#### 2.5.2 Learning rate scheduling

For Faster R-CNN, Cascade R-CNN with ResNet as backbone, we followed the 2× schedule (He et al., 2019), which entailed fine-tuning for 24 epochs with learning rate drop of 10× at epoch 16 and epoch 22.

However, for Faster R-CNN and Cascade R-CNN with the ConvNext-tiny backbone, we extended the training epoch to 36, and decreased the learning rate at epoch 27 and epoch 33 by a factor of 10×.

As for DINO, it was fine-tuned for 24 epoch, with learning rate decay of 10× at epoch 20.

When it comes to the YOLO series and RT-DETR, we adopted the OneCycle learning rate schedule (Smith and Topin, 2017), which is the default schedule in Ultralytics. We used this schedule for fine-tuning over 100 epochs.

#### 2.5.3 Hyper-parameters

For Faster R-CNN and Cascade R-CNN with ResNet as the backbone, we utilized the SGD optimizer with the following hyperparameters: an initial learning rate of 0.02, 500 steps of linear warm-up, weight decay of 0.0001, and a momentum of 0.9.

For Faster R-CNN with ConvNext-tiny as the backbone, we employed the AdamW optimizer with a learning rate of 0.0001, betas set to (0.9, 0.999), weight decay of 0.05, and a decay rate of 0.95 for layer-wise learning rate decay, with 6 top layers.

For Cascade R-CNN with ConvNext-tiny as the backbone, the learning rate was set to 0.0002, and the decay rate for layer-wise learning rate decay was set to 0.7. Other hyperparameters were consistent with Faster R-CNN using ConvNext-tiny.

As for DINO, we used AdamW with a learning rate of 0.0001 and weight decay of 0.0001, clip gradients with a maximum norm of 0.1 and norm type 2. The learning rate for the backbone was set to 0.00001.

Regarding the YOLO series and RT-DETR, we utilized the AdamW optimizer with the following hyperparameters: a max learning rate of 0.000714, initial learning rate factors of 0.1, final learning rate factor of 0.0005, weight decay of 0.937, and beta1 of 0.1. The anneal strategy was linear, with 3 warm-up epochs, an initial warm-up momentum of 0.8, and an initial bias learning rate of 0.1.

All the models were trained on 8 GPUs with a mini-batch size of 2 per GPU. During model validation, confidence score thresholds and IoU thresholds for Non-Maximum Suppression (if the model required NMS) were set to 0.05 and 0.5, respectively. For predictions, these thresholds were adjusted to 0.3 and 0.5.

All unmentioned hyperparameters are set to default values in Pytorch.

#### 2.5.4 Data augmentation

To improve model robustness and increase data diversity, we applied various data augmentation techniques, such as vertical and horizontal flipping, HSV color space enhancement, blur, median blur, and CLAHE. For the YOLO series, we also incorporated mosaic and random affine transformations. A detailed configuration is available in **Table 2**.

**Table 2:** Data augmentation configuration.

Data Aug.	Config(Prob./Frac.)
Horizontal/Vertical Flipping	0.5
HSV-Hue	0.015
HSV-Saturation	0.7
HSV-Value	0.4
RandomAffine	1.0
Mosaic	1 (1-90 epochs), 0(90-100 epochs)
Blur	0.01, limit=(3, 7)
MedianBlur	0.01, limit=(3, 7)
CLAHE	0.01, clip limit=(1, 4), tile grid size=(8, 8)

### 2.6 Metrics for evaluation

We employed four metrics to assess count performance, which include the Root Mean Squared Error (RMSE), the Coefficient of Determination (*R*^2^), Mean Average Precision (mAP@50:5:95), and Average Precision at IoU 50 (AP@50). The definitions of RMSE, *R*^2^, mAP@50:5:95, AP@50 are given in Equations 4-10.


(4) $$RMSE=\sqrt{\frac{1}{n}\sum_{i=1}^{n}{\left(y_{i}-{\hat{y}}_{i}\right)}^{2}}$$

(5) $$R^{2}=1-\frac{\sum_{i=1}^{n}{\left(y_{i}-{\hat{y}}_{i}\right)}^{2}}{\sum_{i=1}^{n}{\left(y_{i}-\overset{¯}{y}\right)}^{2}}$$

(6) $$Precision=\frac{TP}{TP+FP}$$

(7) $$Recall=\frac{TP}{TP+FN}$$

(8) $$IoU=\frac{area\;of\;overlap}{area\;of\;union}$$

(9) $$AP@k=\int_{0}^{1}P\left(R\right)dR,IoU=k$$

(10) $$mAP@50:5:95=\frac{1}{9}\sum_{k\in 50,55\ldots 95}AP@k$$

Where *n* represents the number of test images, *y_i_* denotes the panicle number counted manually, and ${\hat{y}}_{i}$ signifies the panicle number derived from the prediction of YOLOv8-X. *TP*, *FP*, and *FN* denote the number of true positives, false positives, and false negatives, respectively. In this study, *TP* refers to bounding boxes that correctly detected rice panicles. *FP* represents bounding boxes that erroneously identified background regions as rice panicles. *FN* signifies ground truth rice panicles that were missed by the detection algorithm.

### 2.7 Heading-date-related traits extraction

**Figure 4:**
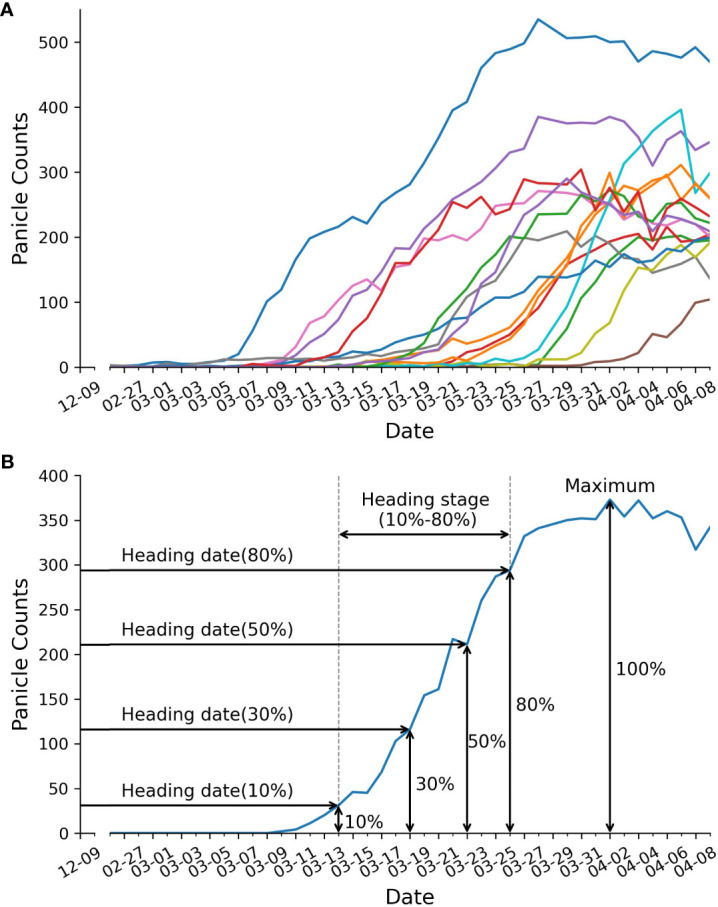
Panicle counts over time. **(A)** displays panicle counts over time for 15 RILs. Each colored line represents the panicle counts for a single RIL. **(B)** depicts trait extraction example, including: heading date, duration of heading stage, and maximum panicle counts.


After counting the number of panicles in each plot, we created growth curves represented the panicle count in each plot over time (**Figure 4A**). These growth curves served as the basis for extracting five static traits and one dynamic trait, as illustrated in **Figure 4B**. The extraction procedure is described as follows: Firstly, we determined the maximum panicle count. Next, we identified specific developmental stages, which correspond to 10%, 30%, 50%, and 80% of the maximum panicle count. For each of these stages, we used Equation 11 to calculate the date at which each stage was reached. The dynamic trait, the heading stage or heading rate, was defined as the difference between the date of reaching 10% of the maximum panicle count and the date of reaching 80% of the maximum panicle count.

(11) $$y\;\text{heading date}=\arg\;\underset{x}{\min}\left(\left|\text{panicle counts of }x-y\times \text{Maximum}\right|\right),y\in \left\{10\%,30\%,50\%,80\%\right\}$$

Where *x* denotes the date.

### 2.8 QTL mapping

The static and dynamic traits were validated through QTL mapping using the UAV-measured heading date-related genetic traits and manually-scored traits collected from RILs. Sequencing and genotyping for the 191 homozygous RILs were conducted using a published pipeline and SEG-MAP (Zhao et al., 2010). Composite interval mapping for QTL analysis was performed using Windows QTL Cartographer version 2.5 (Wang, et al., 2012). The Logarithm of the Odds (LOD) value was calculated to indicate the possibility of QTLs based on likelihood ratio tests.

## 3 Results

### 3.1 Collected 2D aerial images

We used the DJI M300 drone, equipped with the H20 camera, to monitor rice experiments from February 26 to April 9, 2023. During this period, we systematically generated 42 series of 2D aerial images for each experimental plot. As a result of all the flight operations, we produced a substantial 160 GB of high-quality 2D imagery.

### 3.2 Models performance comparison

In order to find the model that best fits panicle detection, we selected several models from three main categories of object detection models.

We trained Faster R-CNN, Cascade R-CNN, YOLOv5, YOLOv8, RT-DETR and DINO with different model sizes and backbones. The performance evaluation was conducted on one main test set and two sub-test sets. These sub-test sets, derived from the main test set, contained early-stage rice panicles and late-stage rice panicles, respectively (refer to **Table 1**).

Our results indicated that the performance of models aligned with our expectations regarding the Average Precision(AP) metric. Models with more parameters and advanced backbones consistently delivered superior results on this metric. Faster RCNN and Cascade RCNN, which employed ConvNext as their backbone, had higher AP values compared to those using ResNet. Similarly, the AP value of the YOLO series showed an increase as the model size grew. Furthermore, YOLOv5-P6, which employed a larger image resolution as input, performed an additional downsampling, and utilized a higher-level feature map, achieved better performance compared to YOLOv5. The situation in the DETR series mirrored that of the R-CNN and YOLO series, with DINO, which used Swim-L as the backbone, achieving the highest AP value among all models.

The AP metric didn’t exhibit a strictly positive correlation with the *R*^2^ and RMSE metrics across various model architectures. This phenomenon was particularly noticeable within the DETR series. For instance, when RT-DETR and DINO-R50 achieved a comparable AP to other models, their *R*^2^ values were significantly lower than those of the YOLO and R-CNN series. DINO-Swim-L, despite attaining the highest AP, only exhibited performance levels on par with the Faster RCNN series in terms of *R*^2^ and RMSE. Surprisingly, RT-DETR-L even yielded a negative *R*^2^ value. After comprehensive consideration of these metrics, our choice for a detector fell on YOLOv8-X. On the test set, early test set, and late test set, its *R*^2^ and RMSE values stood at 0.927, 3.442, 0.963, 2.447, 0.864, and 4.200, respectively. Furthermore, it achieved mAP@50:5:95 and AP@50 values of 0.674 and 0.897 on the test sets.

### 3.3 Time-series image detection

After a comparative evaluation, we employed the YOLOv8-X model for panicle counting. Following the methodology described in the Methods section, we generated curves illustrating panicle counts over time for 15 out of 294 lines (**Figure 4A**) and successfully obtained six traits, comprising five static traits and one dynamic trait (**Figure 4B**). These traits included maximum panicle counts, four heading dates at 10%, 30%, 50% and 80% panicle counts, and the duration of the heading stage (defined as the period between the 80% heading date and the 10% heading date) (**Figure 4B**). Notably, we were able to capture the dynamic trait of heading stage duration, which was previously unattainable through manual phenotype analysis.

**Figure 5:**
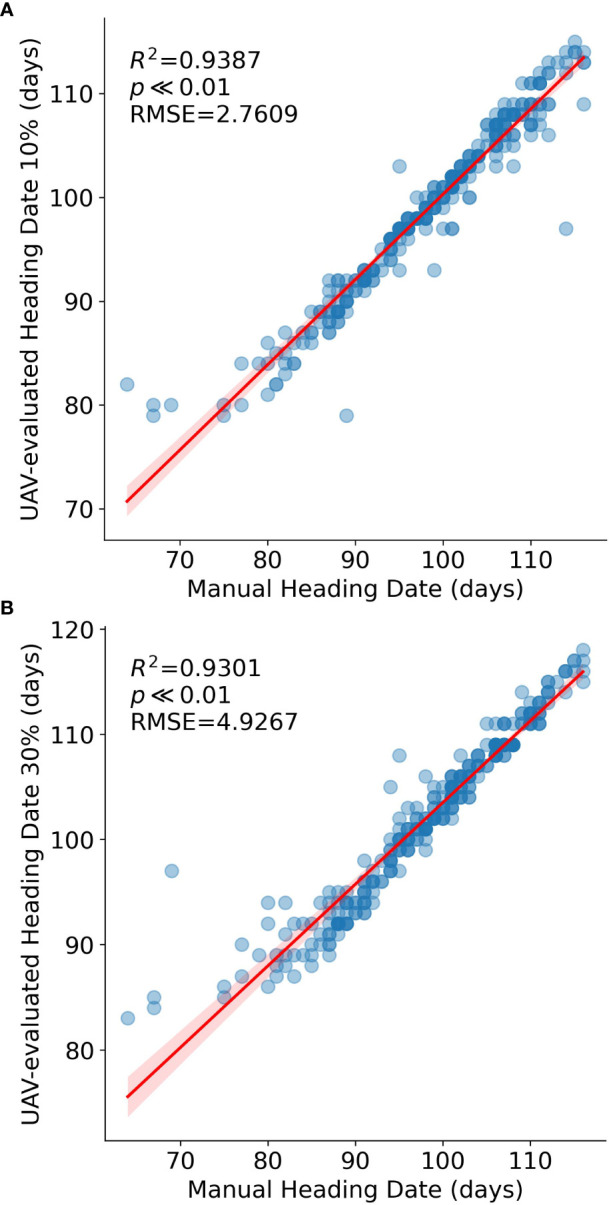
Comparison between Manual and UAV-evaluated heading dates (n=294 RILs). Data points represent single RIL measurements. The red line represents the regression line. **(A)** heading date at 10% panicle counts versus Manual heading date, **(B)** heading date at 30% panicle counts versus Manual heading date.

Moreover, we compared the 10% and 30% heading dates with manually recorded heading dates (**Figure 5**) for validation purposes. The *R*^2^ values for these two developmental stages were 0.9387 and 0.9301, respectively, providing strong support for the validity of our methodology.

### 3.4 QTL mapping using heading-date-related traits

To assess the biological significance of UAV-evaluated traits in genetic mapping studies, we employed a set of 191 homozygous RILs for genetic linkage analysis. The UAV-based evaluation of heading-daterelated traits was utilized to map QTLs within the population. The genetic distance along the x-axis of 12 chromosomes and the LOD (logarithm of odds) value along the y-axis were used for graphical representation. A threshold value of 3.0 (indicated by the red horizontal line) was employed, and known loci were denoted by red arrows.

**Figure 6:**
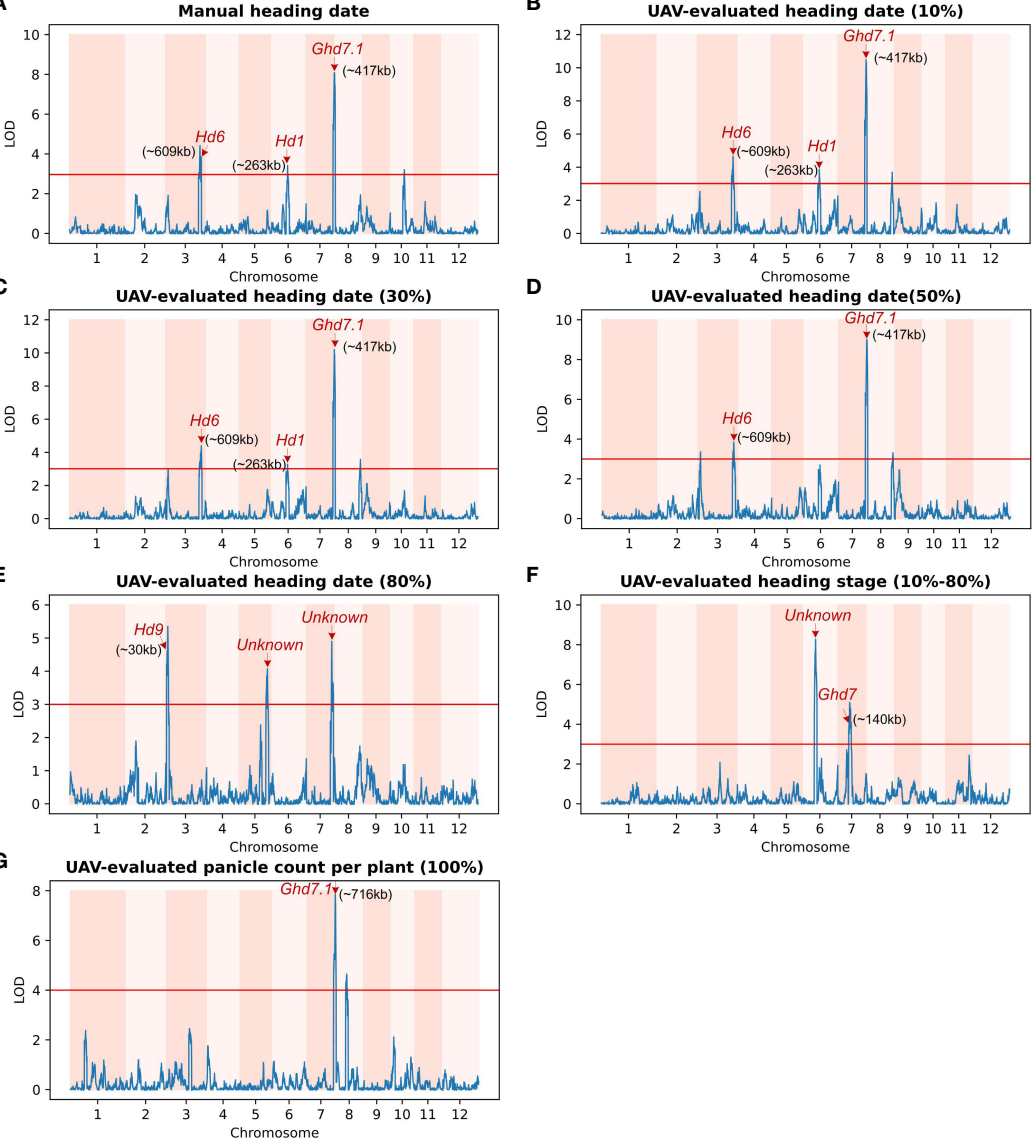
Genetic linkage analysis of various UAV-evaluated heading date related traits and manually recording in a population of 191 homozygous recombinant inbred lines (RILs). Red arrows indicate known genes associated with significant single-nucleotide polymorphisms (SNPs).The x-axis represents the genetic distance of the 12 chromosomes, while the y-axis represents the logarithm of the odds (LOD) value. The red horizontal line indicates the significant threshold set at 3.0. **(A)** QTLs identified using Manual heading date. The identified QTLs are close to the *Hd6* gene (chromosome 3), the *Hd1* gene (chromosome 6) and the *Ghd7.1* gene (chromosome 7). **(B)** QTLs identified using UAV-evaluated heading date at 10% panicle counts. **(C)** QTL for UAV-evaluated heading date at 30% panicle counts. Similar to **(A)**, the QTLs identified using UAV-evaluated heading date at 10% and 30% panicle counts are also located in the vicinity of the *Hd6*, *Hd1*, and *Ghd7.1* genes. **(D)** QTL for UAV-evaluated heading date at 50% panicle counts. **(E)** Three loci associated with UAV-evaluated heading date at 80% panicle counts, including one located near *Hd9* (chromosome 3), and another two significant loci on chromosome 5 and 7 that are not associated with any known gene. **(F)** Two QTLs for UAV-evaluated heading stage (date of 80% - date of 10%). The major QTL is not associated with any known gene, while the other is close to the *GHd7* gene. **(G)** QTL for UAV-evaluated panicle counts per plant. The major QTL co-locates with *Ghd7.1* gene.

Among the traits analyzed, including manual heading date (**Figure 6A**), UAV-evaluated heading date at 10% panicle counts (**Figure 6B**), and UAV-evaluated heading date at 30% panicle counts (**Figure 6C**), we identified three consistent QTLs. Notably, in **Figure 6B**, the most significant QTL (LOD = 10.26) was located on chromosome 7, approximately 417 kb away from the known gene *Ghd7.1*. This gene, as reported by Yan et al (Yan et al., 2013), plays a crucial role in grain productivity and rice heading. The second highest peak, observed using the heading date (10%) trait, was found on chromosome 3 (LOD = 7.3), approximately 609 kb away from *Hd6* (Ogiso et al., 2010), a gene known to regulate rice flowering and dependent on a functional *Hd1* gene. Furthermore, the third highest peak, identified using the heading date (10%) trait, was situated on chromosome 6 (LOD = 3.84), approximately 263 kb away from *Hd1*, a gene responsible for promoting flowering (Zong et al., 2021). In the trait analysis of UAV-evaluated heading date at 50% panicle count (**Figure 6D**), we identified two QTLs located on chromosome 3 and 7, as described above. In **Figure 6E**, we detected a QTL (LOD = 4.58) on chromosome 3, approximately 30 kb away from the *Hd9* gene, which controls rice heading date (Hongxuan et al., 2002).

In addition to static traits, we utilized the dynamic trait, UAV-evaluated heading stage (from 10% panicle counts to 80% panicle counts), to map QTLs, resulting in the identification of two QTLs (**Figure 6F**). The first QTL was located approximately 140 kb away from *Ghd7* (LOD = 3.99), a gene known to delay heading under long-day conditions while increasing plant height and panicle size (Hu et al., 2020). The second QTL was found approximately 7.1 Mb along chromosome 6 (LOD = 8.24) and was not associated with any known gene. Subsequently, we conducted a QTL mapping using UAV-evaluated panicle count per plant (**Figure 6G**), we identified a QTL located approximately 706 kb away from the known gene *Ghd7.1*. A comprehensive list of all QTLs identified through QTL mapping is provided in **Table 3**.

**Table 3:** Quantitative trait loci (QTLs) for heading date, heading stage, and panicle count identified in 191 rice RILs using manual and UAV phenotyping.

Traits	Chr	Peak gent. Pos.	IRGSP1.0 (Mb)	LOD	R^2^	Add.	Genes
Manual heading date	3 6 7	271.51 124.31 202.91	30.9 9.6 29.2	3.8 3.4 8.0	5.8% 4.9% 12.6%	2.52 2.47 3.62	*Hd6*(31.51M) *Hd1*(9.34M) *Ghd7.1*(29.62M)
UAV-evaluated heading date(10%)	3 6 7	271.51 124.31 202.91	30.9 9.6 29.2	4.6 3.8 10.3	6.4% 5.2% 15.3%	2.14 1.94 3.35	*Hd6*(31.51M) *Hd1*(9.34M) *Ghd7.1*(29.62M)
UAV-evaluated heading date(30%)	3 6 7	271.51 124.31 202.91	30.9 9.6 29.2	4.4 3.2 9.9	6.0% 4.4% 14.7%	2.00 1.74 3.20	*Hd6*(31.51M) *Hd1*(9.34M) *Ghd7.1*(29.62M)
UAV-evaluated heading date(50%)	3 7	271.51 202.91	30.9 29.2	3.8 8.9	5.5% 13.8%	1.73 2.78	*Hd6*(31.51M) *Ghd7.1*(29.62M)
UAV-evaluated heading date(80%)	3 5 7	8.61 218.01 182.21	1.3 26.5 26.2	4.6 4.1 4.9	7.2% 6.8% 7.7%	1.71 1.77 1.82	*Hd9* (1.27M) Unknown Unknown
UAV-evaluated heading stage(10%-80%)	6 7	97.61 79.61	7.1 9.3	8.2 4.0	13.9% 6.5%	-1.69 1.73	Unknown *Ghd7*(9.15M)
UAV-evaluated Panicle Count per plant	7	201.31	28.9	7.8	12.1%	-1.85	*Ghd7.1*(29.62M)

## 4 Discussion

This study underscores the potential of integrating UAV imagery and object detection models for high throughput, field-based phenotyping of agronomic traits in rice. By harnessing the capabilities of the M300 UAV, equipped with an H20 camera, we are able to swiftly capture images for 294 RILs. This operation, requiring only a single operator, can be completed within a two-hour timeframe. The application of the cutting-edge YOLOv8-X model on UAV-acquired images with a simple image process pipeline, enables the rapid extraction of panicle count data at various developmental timepoints. Additionally, our semi-automatic labeling pipeline reduces the labor cost needed for training a usable object detection model. In summary, our comprehensive approach facilitates cost-effective analysis of six crucial heading-date related traits. Without this approach, a comparable scale of analysis would require a prohibitively extensive investment of time and labor for manual measurements.

Indeed, the application of deep learning to plant phenotyping is becoming increasingly common today. There are several works that focus on panicle detection and heading date estimation using deep learning methods. For instance, in (Zhou et al., 2019), the authors proposed an improved R-FCN for detecting panicles from different stages of rice growth, achieving a precision of 0.868 on their held-out test set. Taking into account the popularity and representativeness of the models, we have not tested the model on our dataset.

Teng integrated several object detection models, such as Faster RCNN and YOLOv5, into a single web platform. These models were used to detect panicles and calculate the panicle number per unit area (PNpM2). They also proposed a tailored YOLOv5 model called Panicle-AI, which has a better AP@.5 of 0.967 than the original YOLOv5 (0.954) on their test set (Teng et al., 2023).In this paper, we not only obtained panicle counts per plant, similar to the panicle number per unit area, but also extracted five additional traits related to heading dates based on time-series images.

Instead of focusing on model modification, some researchers direct their attention to the improvement of NMS, an important part of the objective detection algorithm. This has been proven to perform better in removing redundant bounding boxes under crowded conditions, thereby improving detection accuracy. In our method, we used standard NMS; therefore, there may be an improvement in accuracy when using their method (Wang et al., 2022).

Another work also focuses on the heading date, but uses a paradigm proposed in 2013 (Girshick et al., 2014), which was no longer used within two years. They concentrate on detecting flowers to estimate the heading date, and their method has not been tested on a large scale population (Desai et al., 2019).

Some other methods do not use object detection, simply employing backbones like ResNet for regression tasks. Guo et al. used a modified DenseNet to directly predict the panicle ratio from images. They achieved an *R*^2^ of 0.992 in their estimation of the heading date. However, their labeling process requires a significant amount of labor to count the number of panicles and the number of tillers via a field survey. Our method requires much less labor, estimating different stages of the heading date based on the panicle number, and further validating through QTL mapping (Guo et al., 2022).

Returning to our result, the high R-squared achieved by the model in panicle counting demonstrates a strong alignment between model predictions and ground truth data. However, it’s worth noting that the metric AP, typically employed to assess detection models, exhibits a negative correlation with some models, and it may not comprehensively represent model performance for agricultural tasks such as panicle counting. In the future, adopting metrics like RMSE and R-squared, computed against the ground truth panicle counts for model selection, or devising a tailored loss function that accommodates counting errors, could potentially enhance performance in the panicle counting task (Huang et al., 2016).

Nevertheless, the associations established between the traits extracted from UAV imagery and genetic markers affirm the reliability of our phenotyping methodology. This analysis revealed numerous noteworthy QTLs, encompassing both newly discovered loci and loci corresponding to well-known heading-date genes. Notably, the QTLs identified for the 10% and 30% heading dates coincided with those determined through manual heading date assessment, further validating the effectiveness of our UAV-based phenotyping approach. Particularly, in the later stage (heading date 80%), we unveiled new QTLs. Of significant importance is the successful capture, for the first time, of the dynamic trait—the duration of the heading date, which unveiled previously undiscovered QTLs. These novel QTLs suggest the involvement of additional candidate genes that potentially regulate variations in heading-date-related traits.

To advance this research, ongoing refinement of the detection models is essential to maximize accuracy and generalizability. The semi-automatic annotation workflow introduced in this study has the capacity to streamline the labeling of field images, leading to the creation of more extensive training datasets. This, in turn, holds the promise of progressively boosting model performance in a cost-effective manner. In summary, this study underscores the powerful synergy between UAV and computer vision technologies as a promising framework for expediting genetics research and breeding programs focused on crucial agricultural traits in rice and other crops.

## Data availability statement

The raw data supporting the conclusions of this article will be made available by the authors, without undue reservation.

## Author contributions

RC: Writing – original draft, Writing – review & editing, Data curation, Formal Analysis, Methodology. HL: Writing – original draft, Writing – review & editing, Data curation, Formal Analysis, Methodology. YW: Writing – review & editing, Data curation. QT: Writing – review & editing. CZ: Writing – review & editing. AW: Writing – review & editing. QF: Writing – review & editing. SG: Writing – review & editing. QZ: Writing – review & editing, Conceptualization, Funding acquisition, Supervision. BH: Writing – review & editing, Conceptualization, Supervision.
